# The impact of the treaty basis on health policy legislation in the European Union: A case study on the tobacco advertising directive

**DOI:** 10.1186/1472-6963-8-77

**Published:** 2008-04-08

**Authors:** Sandra Boessen, Hans Maarse

**Affiliations:** 1 Department of Health Organization, Policy and Economics, Faculty of Health, Medicines and Life Sciences, Maastricht University, P.O. Box 616, 6200MD Maastricht, The Netherlands

## Abstract

**Background:**

The Europe Against Cancer programme was initiated in the late 1980s, recognising, among other risk factors, the problematic relationship between tobacco use and cancer. In an attempt to reduce the number of smokers in the European Community, the European Commission proposed a ban on tobacco advertising. The question of why it took over ten years of negotiating before the EU adopted a policy measure that could in fact improve the health situation in the Community, can only be answered by focusing on politics.

**Methods:**

We used an actor-centred institutionalist approach, focusing on the strategic behaviour of the major actors involved. We concentrated our analysis on the legal basis as an important institution and evaluated how the absence of a proper legal basis for public health measures in the Treaties influenced policy-making, framing the discussion in market-making versus market-correcting policy interventions. For our analysis, we used primary and secondary sources, including policy documents, communications and press releases. We also conducted 9 semi-structured interviews.

**Results:**

The ban on tobacco advertising was, in essence, a public health measure. The Commission used its agenda-setting power and framed the market-correcting proposal in market-making terms. The European Parliament and the Council of Ministers then used the discussion on the legal basis as a vehicle for real political controversies. After adoption of the ban on tobacco advertising, Germany appealed to the European Court of Justice, which annulled the ban but also offered suggestions for a possible solution with article 100a as the legal basis.

**Conclusion:**

The whole market-making versus market-correcting discussion is related to a broader question, namely how far European health regulation can go in respect to the member states. In fact, the policy-making process of a tobacco advertising ban, as described in this paper, is related to the 'constitutional' foundation of health policy legislation in the Community. The absence of a clear-cut legal basis for health policies does not imply that the EU's impact on health is negligible. In the case of tobacco-control measures, the creative use of other Treaty bases has resulted in significant European action in the field of public health.

## Background

Since the launch of the Europe against Cancer (EAC) programme in 1987, diverse tobacco control measures have been introduced: a ban on tobacco advertising on television (89/552/EEC), tobacco labelling (89/622/EEC and 92/41/EEC), tar maximums for cigarettes (90/239/EEC) and minimum tax levels for tobacco products (92/12/EEC, 92/79/EEC and 95/59/EEC) [1]. Most contentious was, without any doubt, the ban on tobacco advertising. Its adoption in 1998 took almost ten years of negotiation, but even after the final decision in the European Parliament (EP) and the Council of Ministers, the process did not end there. In 2000, the European Court of Justice (ECJ) annulled the directive after an appeal made by Germany and a few other opponents. Following this ruling, the Commission came up with a new proposal in 2001 that was approved by the EP and the Council in 2003. Despite the restricted scope of the new directive, Germany appealed again.

The ban on tobacco advertising proved politically controversial. For instance, Denmark argued that it conflicted with freedom of speech, whereas other countries including Germany, the United Kingdom (UK), the Netherlands as well as Denmark considered regulations on tobacco advertisements a national competence. These countries had a tradition of minimal state intervention in the private sphere of consumption and preferred voluntary agreements with the tobacco industry [1]. Germany even declared the ban unconstitutional. At a deeper level, however, there was a more fundamental conflict between the interests of public health and economic interests. Advocates of a ban on tobacco advertising argued that it would reduce the number of smokers, since advertising promoted the idea of tobacco as a legitimate and socially acceptable product. Opponents of the ban put forward the argument that advertising did not affect the number of smokers. According to them, advertising was necessary for companies to encourage brand building [2]. A ban would also hurt the tobacco and advertisement industry and, therefore, have a negative impact on the labour market. The costs of harmonisation would be centred on Germany, the UK and the Netherlands as the major tobacco manufacturing countries. Thus, there was not only controversy regarding the welfare aspects of the ban (does it generate health gains?), but also, and perhaps more importantly, about its distributional economic impact.

In this article, we analyse the policy-making process of the tobacco advertising ban, focusing on the legal basis. Every piece of legislation enacted by the Community must have a correct legal basis in the Treaties. The Commission framed the ban as a market-making policy intervention directed at removing barriers to foster market competition and free movement. Opponents of the ban claimed that it was not a market-making, but a market-correcting policy intervention, for which there was no legal basis. In fact, different actors used the legal basis as a vehicle for real political controversies. In order to understand why it was so difficult to adopt a policy measure that could in fact improve the health situation in the European Union (EU) [2-4], we claim it is necessary to focus upon the political sphere [5]. We will show that the political controversy regarding the tobacco advertising ban, to a great extent, concentrated upon the question of whether or not it had a correct legal basis. Although our main focus concerns the annulled Directive 98/43/EC, we will also provide an analysis of the discussion on the legal basis for Directive 2003/33/EC.

## Methods

Our analysis is based upon the framework of actor-centred institutionalism [6]. The starting-point of this type of policy analysis is that institutions – defined as the set of 'formal and informal rules of the game that structure the course of action that actors may choose' [6] – influence policy-making processes and their outcomes. Institutions do not have a determinative impact upon the activities of the actors involved. They constrain or facilitate strategic options, but do not determine them [7]. In other words, institutions only create a set of strategic options for actors, but how they use them depends upon their preferences and their assessment of the consequences of the strategic options available. In fact, one may conceptualise the use of institutions as a strategic game in itself [6].

From a problem-solving perspective, institutions are important. They have a strong impact on whether or not an agreement can be reached and, therefore, on the problem-solving capacity of an institutional setting. For instance, if agreement between actors is difficult, the rule of unanimity is likely to block the policy-making process, because each actor has a veto position. Voting by qualified majority would offer a way out of this deadlock. In a similar way, one can conceptualise the introduction of the co-decision procedure in the Treaty on European Union (TEU) as an important institutional change. It implied a significant alteration of the rules of the game governing the relationships between the Council, the Commission and the EP with far reaching consequences for European policy-making [8]. In this article, we analyse how the institutional structure affected the political controversy on the ban on tobacco advertising. What happened within member states falls outside the scope of this article, as does the (strategic) behaviour of interest groups [9].

For this study, an extensive search and analysis were carried out of both data from primary and secondary sources. Our data collection started with a literature review of EU policy-making in general, as well as policy-related studies. This included the archives of the Dutch Ministry of Health and policy documents originating from the Commission, the Council and the EP. We also analysed documents from the European Bureau for Action on smoking Prevention and the European Cancer Leagues. Tobacco industry documents were available on the internet as a result of the 1998 Master Settlement Agreement between 46 United States state attorneys general and the industry. Publications from the press agency 'Agence Europe' also proved a useful source of information.

During the period between April 2005 and March 2006, in-depth, taped, semi-structured interviews were held with nine persons who were all intensively involved in the policy-making process. We used the results of our document analysis to identify persons who were asked for an interview. The 'snowball effect' provided us with other relevant actors (see appendix). We began each interview with a very general question: 'What was your role in the negotiating process concerning....?' This allowed the interviewees to give an open answer before specific questions related to strategic behaviour were asked. The fact that the revised Directive 2003/33/EC (adopted in 2003) was then still pending in the ECJ, proved a complicating factor.

### Discussion: market-making versus market-correcting interventions

Policy interventions with respect to the European market can be divided into two analytical categories: market-making and market-correcting interventions. Market-making interventions are directed at the removal of barriers to trade and competition, including the abolishment of tariffs and other quantitative restrictions, as well as the establishment of common product standards. Another purpose of these interventions is to establish a common level playing field for all market players. From the market-making perspective, all factors that distort the market process must be removed. Market-correcting interventions have a restrictive impact on the domain of competition. Policy-makers implement such interventions in order to avoid market effects they consider deviant or anomalous, such as ethical reasons or considerations related to social welfare and public health. A ban on market instruments, such as advertising, can also be interpreted as a market-correcting intervention if that intervention aims at avoiding unwanted market effects. The distinction between market-making and market-correcting policy interventions should be understood as an analytical one. In fact, many policies combine aspects of both and a clear-cut distinction does not always exist. Yet, it is useful for our research topic to differentiate between interventions directed at the creation of a market and interventions that pose restrictions to the market process for moral, social or other reasons.

How did the Commission's proposal for a ban on tobacco advertising fit into the distinction between market-making and market-correcting? The Commission based the tobacco advertising proposal on article 100a, arguing that its aim was to ensure the free movement of newspapers, magazines and other publications that was hindered by differences in national regulatory regimes.

Yet, the Commission's argument was artificial, because it was evident that the whole idea of a European ban on tobacco advertising originated from the 'Europe against Cancer' programme [10,11]. In our view, the tobacco advertising ban should be understood as a market-correcting intervention rather than a market-making one. It was not directed – or at least not primarily – at the removal of trade barriers, or the establishment of a common level playing field, but instead at the promotion of public health. What the Commission did was to *frame *an essentially market-correcting policy intervention as a market-making one. This observation raises important questions. Why did the Commission do so? How did the controversy about the true nature of the ban influence the strategies of the major players involved? How did the annulment of the ban by the ECJ impact upon the course of the policy-making process? These questions will be dealt with in the next sections.

## Results

### I. The legal basis: political interpretation

Within EU policy-making, an important institution is that policy interventions must have a correct legal basis. According to the ECJ, the choice of the legal basis should be a matter of principle and not a matter of political pragmatism [12]. Was this indeed the case? In this section we will show that the Commission's strategic choice for article 100a as the proposal's legal basis generated much political controversy in the Council and the EP.

#### The European Commission: in search for a legal basis

When the Commission came up with its first proposal for a directive on tobacco advertising in 1989 [13], it had a limited scope. The proposal did not impose a ban, but only included *restrictions *to advertising. Although the proposal was clearly motivated by the ambitious goals of the EAC programme, the Commission's principal argument was that the removal of all obstacles to trade by 1992 required the harmonisation of national regulations at the European level, including those on tobacco advertising. Differences in national regulations hindered the free movement of tobacco advertising publications. Whereas Portugal and Italy had implemented a total ban, Belgium, Ireland and Luxembourg had only banned advertising in publications directed at young people. Spain, Denmark, the UK and the Netherlands opted for partial restrictions based on legislation, or voluntary agreements with the tobacco industry.

The 1989 'restricting' proposal: Advertising for tobacco products in the press and by means of bills and posters should carry health warnings. The content of the advertising message is restricted to information about the product (tar, nicotine yields etc.) and a presentation of its packaging. Advertising in publications intended for people under 18 years is not permitted. Indirect advertising is prohibited. Member states can adopt additional restrictions. Publications or the display of bills which comply with this directive cannot be prohibited.

After the Commission had tabled the 1989 proposal, the EP gave its opinion during first reading under the cooperation procedure. The Committee on the Environment, Public Health and Consumer Protection (the ENVI Committee) advised the plenary to amend the proposal by asking for a *total ban*.

During the plenary meeting, most members of the Parliament (MEPs) emphasised the public health aspect of the Commission's proposal and regarded the suggested amendments for a total ban as an improvement for public health. The market-making argument regarding establishing an internal market for tobacco advertising hardly played a role. Only the European People's Party (EPP) argued that a ban lacked a legal basis in the Treaty and, therefore, fell outside the scope of the Community.

The Commission accepted the Parliament's principal amendment for a ban on tobacco advertising in its proposal tabled in 1991 [14]. Policy developments in some member states had encouraged the Commission to replace its proposal for restrictions to tobacco advertising with a ban on tobacco advertising. Public regulations in Belgium and Greece had become more restrictive and France had even adopted a ban on tobacco advertising.

The 1991 'banning' proposal: Direct as well as indirect advertising for tobacco products are banned within the Community. Free distribution of tobacco products is not allowed. Member states can authorise advertising within tobacco sales outlets, provided it is not visible from outside the premises.

The Commission's original proposal restricting advertisements (1989) and its new proposal banning tobacco advertising (1991) were both based upon article 100a. The Commission stipulated that the main goal of these proposals was to reinforce the functioning of the internal market, although it was clear right from the beginning that they also served a public health purpose. One may argue that the market-making argument made by the Commission could be justified under the condition that the proposal only contained restrictions to tobacco advertising. The diversity in national regulations on tobacco advertising could indeed hinder international trade. Following this reasoning, harmonisation by means of adopting restrictions to advertising would improve the free movement of publications.

However, when the Commission introduced a ban on tobacco advertising, its argument became seriously flawed. As one of our interviewees explained, "If [there are] advertisements for tobacco in a German cinema, what does it have to do with the internal market? What about posters in Bonn or Berlin? [They have] nothing to do with the internal market" (*#1, German civil servant*). The proposal's principal purpose became combating smoking, instead of harmonising national regulations.

Why then, did the Commission choose article 100a as the legal basis for the 1991 proposal? Prior to the TEU, the Community lacked any specific authority to develop policies that had public health protection as their 'principal or sole justification'. However, 'the Single European Act (SEA) made it possible to piggy-back health considerations onto the development of the single market' [15], because it recognised health as a dimension of economic integration. The vague language of the SEA enabled the Commission to take action in the public health sphere. The formulation of the proposal required creative wording, due to the lack of an explicit public health competence. The choice for article 100a implied that the principal objective had to be the establishment and functioning of the internal market. The Commission also referred to paragraph 3, of article 100a, according to which, the Commission should take a high level of protection of health as a basis for its proposals concerning the establishment and functioning of the internal market. However, the Commission's interpretation of this paragraph was ambiguous. It required the Commission to take health protection into account when developing policy interventions directed at the removal of obstacles to trade. It did not imply that 'a high level of protection of health' could be taken as the main objective of a policy intervention. In other words, 'a high level of protection of health' should be interpreted as a secondary policy, not as a policy objective in itself.

In fact, the Commission believed it had no real alternatives to article 100a. It took until 1993, before the Community was given a legal competence in the field of public health. Article 129 of the TEU (now article 152) gave the EU a mandate to 'encourage cooperation between member states and, if necessary, lend support to their actions'. However, article 129 excluded harmonisation of laws and regulations in member states and, therefore, could never provide a legal basis for the tobacco advertising ban. The only alternative was article 235 (now article 308), which provided the Commission with the room to act in circumstances where action by the Community was necessary, even though no legal basis existed. However, article 235 required decision-making by unanimity in the Council and decreased the role of the EP, which now only needed to be consulted. Given the diverging preferences in the Council, the Commission did not consider this feasible. Article 100a was considered to be the only option, because decisions based upon it could be taken by qualified majority voting (QMV). Thus, the choice for article 100a was a strategic one. It reflected the Commission's assessment that the 'article 235 route' was likely to fail, because of the impossibility of reaching political consensus on a ban.

The Commission's strategy came at a price. A key problem was that an essentially market-correcting policy intervention had to be framed in terms of a market-making intervention. This problem proved a source of fundamental tensions that played a significant role during policy-making.

#### The Council of Ministers

The QMV rule enabled countries opposing the ban to block agreement for many years. Germany, the UK, the Netherlands, Greece and Denmark renounced the ban "for purely economic reasons" (*#1, German civil servant*). The interests of the tobacco and advertising industry apparently prevailed over public health interests.

The Commission's choice for article 100a as the legal basis of the tobacco advertising ban influenced negotiations in the Council. The Council's legal service concluded that article 100a provided an appropriate legal basis for the proposal on *restricting *tobacco advertising. Differences between national regulations caused a distortion of competition. Resorting to restrictions on tobacco advertising, instead of imposing a ban, would leave media free to circulate in the internal market under given conditions.

The legal service also examined the proposal for a *ban *on tobacco advertising, concluding that the ban 'cannot be regarded as having the effect of harmonizing national provisions relating to the establishment and functioning of the internal market. The provision covers purely domestic situations and is designed to protect public health'. In other words, the legal service held the opinion that the ban could not be based on any Treaty article.

This opinion was obviously good news for the opponents of the ban, using it as an important argument to obstruct its acceptance. For example, the Belgian Minister of Health exploited the conclusions of the Council's legal service to distort the Health Council meeting – undermining her role as president to facilitate the negotiating process during the second half of 1993. In fact, the Belgian government was in favour of the proposal, but the Belgian Health Minister personally did not show any commitment. The discussion in the health working group was based on an informal paper, which proposed a ban, but delayed its implementation until 1998. The Belgian Health Minister had not approved the paper and had no intention of pursuing the dossier. However, the Commission decided to reinstate the item on the Council's agenda [16]. During the following Council meeting, the Belgian Minister cited the opinion of the legal service and returned the proposal to the working group without even a 'tour de table' [17].

Germany tabled a compromise proposal in 1994 during its presidency, limiting the scope of the original proposal to cross-border advertising. The German government held that this was the only possibility under article 100a. Indirect advertising and sponsoring were excluded, thus reducing the scope of the proposal. The UK and the Netherlands had principal objections. They maintained their earlier position that tobacco advertising regulation at the European level conflicted with article 100a and the principle of subsidiarity.

An important event took place in May 1997, when the new UK government rejected all the earlier fundamental objections of the previous government. A similar development occurred in the Netherlands. In 1993, the Dutch government decided to reconsider its point of view in case its vote was decisive, which after the policy change by the UK, was indeed the case. Only a few days before the Health Council meeting in December 1997, the Dutch government decided to vote in favour of the ban. It had always used the ambiguity of the legal basis as a reason to oppose the ban, referring to the opinion of the Council's legal service. Yet, in the final negotiating stage, it reasoned that the legal service of the Commission had expressed a contradictory view. The Dutch Health Minister told the national Parliament that the issue of the legal basis was for the ECJ to sort out.

Thus, member states in favour of the ban on tobacco advertising based their position on the belief that further restrictions to tobacco advertising were required to reduce the harmful effect of smoking on public health. Member states opposing the ban gave priority to their economic interests. The controversy over the legal basis and the opinion of the Council's legal service offered them an opportunity to renounce the ban and obstruct the policy-making process without the need to refer to their economic interests. "Most legal arguments, especially those regarding competence, were just smoke screens for countries who wanted to block the ban" (#*1, German civil servant*).

#### The European Parliament

Whereas the legal basis of the original proposal restricting tobacco advertising was hardly an issue in the EP, it became a major point of discussion during the debate on the proposal for the tobacco advertising ban. The ENVI Committee, which was responsible for the report, supported the proposal. The rapporteur explicitly expressed his support for the Commission's choice of article 100a as the proposals' legal basis.

In February 1992, the EP plenary gave its opinion on this issue [18]. The EPP and the Economic and Monetary Affairs and Industrial Policy Committee tabled amendments to change the legal basis, seeking to replace article 100a with article 235. All MEPs knew that under article 235, decisions had to be made by unanimity. The rapporteur from the ENVI Committee called this 'a deceitful attempt to sweep the text from the table' [19]. Despite the expressions of doubt regarding the legal basis, the plenary rejected the motion to replace article 100a with article 235 (150 votes in favour; 123 against; and 12 abstentions). The head of the ENVI Committee stated that 'the people who wanted to change the legal basis lost. Now what we are seeing is (...) an attempt to delay the proceedings. This is plain filibustering and it is a plain abuse of the democratic process' [20].

After the Council reached its common position in 1997, the EP had to give its opinion in its second reading. In a final attempt to obstruct the adoption of the ban on tobacco advertising, opponents once again concentrated on amending the legal basis. Many documents addressing the legal basis of the ban circulated in the EP. For example, the confederation of European Community Cigarette Manufacturers (CECCM) published a report declaring that the EP was permitted to, and should discuss and amend the legal basis [21]. The International Union against Cancer and the Association of European Cancer Leagues framed the ban as a market-making intervention, stating that article 100a was correct and that differences between member states in regulation of advertising and sponsorship impeded the proper functioning of the internal market [22].

Several (mostly German) MEPs started to raise questions about the legal basis of the Council's common position. For example, Schleicher (EPP, Germany) suggested article 129 as the proper legal basis, since it was generally acknowledged that the directive pursued a health policy objective. In its answer, the Council said that it 'took great pains to produce a text which took into account both the efficient operation of the internal market, and the requirements of ensuring a high level of health protection. (...) The Court of Justice is competent to interpret the measures and to ensure that they comply with the Treaty' [23]. Another example was the question to the Council issued by Jackson (EPP, UK), focusing on the opinion of the Council legal service. '(...) Could the Council explain how the provisions of the current proposal are so different from the 1992 draft as to make the legal service's opinion no longer applicable?' The Council responded that the legal service assisted the Council in its deliberations by providing independent legal advice, which was only an *internal *document [24]. Finally, Florenz (EPP, Germany) asked the Commission what justified its choice for article 100a as the legal basis. The Commission answered that it considered the legal basis sufficient and appropriate [25].

In March 1998, the EP's legal service confirmed the legal basis of the common position. It did not question the Community's competence to rule on the matter. On the contrary, it felt that action at European level was necessary, but suggested that the legal basis could be strengthened by complementing article 100a with article 57 paragraph 2 (now article 47(2)) and article 66 (now article 55). The latter empowered the Council to adopt directives in order to facilitate the exercise of freedom of establishment, and freedom to provide services [26].

The ENVI Committee delivered its opinion in April 1998 [27]. The Committee had heard an oral statement from the Legal Affairs Committee, which had issued a report on its own initiative disputing the competency of the EU to adopt a ban on tobacco advertising. By a mixed view (12 votes in favour; 7 against and 3 abstentions), it concluded that the EU had no legal basis on which to act, because there was no distortion of competition, but only the abolition of an activity. However, the Parliaments' legal service insisted on article 100a as the correct legal basis. Considering the EP's legal service as a body of technical experts instead of politicians who populated the Legal Affairs Committee, the proposal based on article 100a received a go-ahead from the ENVI Committee.

In May 1998, the ban on tobacco advertising was debated in the EP plenary [28], which was divided in two groups. One group was committed to public health, the other to economic interests. The plenary debate again focused on the legal basis. Several MEPs argued that the directive had the correct basis. 'The legal services of Parliament, the Commission and the Council say that article 100a is the correct basis. It is clearly an issue of the internal market'.

Opponents claimed that 'since Maastricht, there has been article 129 which calls on us to promote public health and the route for those who want to ban tobacco advertising is article 129 of the Treaty.' One MEP argued that 'the legal instruments being advanced by the tobacco industry through a skilfully orchestrated lobbying campaign seem intended to evade the debate on the substance of the matter by replacing it with a debate on procedure. (...) Let the Court of Justice decide on the issue of the legal basis, if it is referred to them. That is their right'. The discussion on the legal basis was also called subterfuge: 'When people say we should use article 129, they forget that, according to that article, it is not possible to adopt measures to harmonise the member states' legal and statutory provisions. So it is just a subterfuge, so much so, that we think there has been an attempt to devalue the position of the Council'.

Despite the discussion on the legal basis, the EP voted in favour of the Council's common position in May 1998. Directive 98/43/EC was finally adopted in July 1998.

### II. The legal basis: juridical interpretation

Germany had blocked the proposal for a tobacco advertising ban from the very beginning for economic reasons. After having been outvoted in the Council, it decided to appeal to the ECJ. In addition to the German government, Salamander AG (Germany), owning the Camel Boots trade mark, Una Film (Austria), distributing cinema tobacco advertising films, Alma Media Group (Greece), selling advertising space in public places, and Davidoff (Switzerland), holding the Davidoff trade mark for tobacco products as well as products outside the tobacco sector, also challenged the tobacco advertising directive before the ECJ. However, the Court of First Instance dismissed their cases, not recognising them as interested parties [29].

If the directive would have been adopted by unanimity, one may assume that no government would have an interest in challenging it. Given the fact that only interested parties, (those whose legal situation is influenced directly by the consequences of the directive and the institutions which have given birth to the directive), can bring proceedings, the list of potential plaintiffs is more narrow under unanimity. Thus, QMV increases the chance of litigation before the ECJ, as member states opposing adoption may seek to enforce their point of view by judicial means. The decision of the German government to challenge Directive 98/43/EC created a new institutional setting with the ECJ as arbiter. The interaction mode thus shifted from joint decisions under QMV, to hierarchical direction, centralising competences at supranational level through an ECJ ruling [30,31].

#### The European Court of Justice ruling on Directive 98/43/EC

The debate thus continued via another route. The German government put forward several reasons why the EU was not competent to adopt the measure [32], with its main argument based on an inappropriate legal basis. Following its argument that tobacco advertising is essentially an activity of which the effects do not extend beyond the borders of individual member states, differences in national legislation did not result in distortion of competition. In fact, a ban would allegedly counteract trade instead of facilitating it, by creating new obstacles that did not exist previously.

The Commission, the EP and the Council, joined by France, Finland and the UK, argued that cross-border trade in services and goods in tobacco advertising existed. A Community-wide measure was required because differences in national regulations could be regarded as an obstacle to free trade. They maintained that article 100a also permitted internal market regulation without a liberalising effect: 'the power conferred on the Council by that provision is not necessarily concerned with the liberalisation of trade, but rather with market regulation. That explains why it has been possible for directives containing certain prohibitions to be adopted on the basis of article 100a' (para 45). The Commission also argued that there was, in fact, a real distortion of competition. 'Because of existing differences in legislation, the potential profit of advertising agencies differs according to the (...) market in which they carry on business' (para 51). According to the defendants, the essential factor in assessing the choice of the legal basis was the text of the measure in question. They argued that human health was one of the directives' objectives, but not the principal one. 'The emphasis on public health protection in the directive can be explained by the fact that it constituted the main (...) objective of the national measures being harmonised, but, in the context of harmonisation, it became a secondary objective' (para 56). Finally, all defendants referred to paragraph 3 of article 100a: '(...) a broad prohibition on tobacco advertising derives from the obligation imposed by article 100a(3)' (para 57).

The ECJ stated that the explicit exclusion of harmonisation in article 129 meant the directive could not be based on that particular provision. 'But that provision does not mean that harmonising measures adopted on the basis of other provisions of the Treaty cannot have any impact on the protection of human health. Indeed, the third paragraph of article 129(1) provides that health requirements are to form a constituent part of the Community's other policies. Other articles of the Treaty may not, however, be used as a legal basis in order to circumvent the express exclusion of harmonisation laid down in article 129(4) of the Treaty' (para 78–79). 'Provided that the conditions for recourse to articles 100a, 57(2) and 66 as a legal basis are fulfilled, the Community legislature cannot be prevented from relying on that legal basis on the grounds that public health protection is a decisive factor in the choices to be made' (para 88). The ECJ therefore had to examine whether the directive actually contributed to eliminating obstacles to the free movement of goods and services, and removing appreciable distortions of competition.

With regard to the elimination of obstacles to free movement, the Court ruled that the directive did not improve the functioning of the internal market. The prohibition imposed by the directive was too general because it concerned all forms of advertising. Some forms, in particular, static advertising media, were not cross-bordering in nature.

On what concerns elimination of distortion of competition, 'in examining the lawfulness of a directive adopted on the basis of article 100a of the Treaty, the Court is required to verify whether the distortion of competition, which the measure purports to eliminate is appreciable. In the absence of such a requirement, the powers of the Community legislature would be practically unlimited' (para 106). In this case, the Court argued that the distortions were not considered to be sufficiently appreciable to accept an absolute prohibition of advertising and sponsorship. Based on these findings, the ECJ annulled the directive. Traditionally, the main way in which the Court influenced the development of the Community has been through an expansive interpretation of Community competences. It was the first time the ECJ ruled that the EU did not have the competence to adopt a measure [12].

#### Directive 2003/33/EC: banning cross-border advertising and sponsorship

Interestingly however, the Court went a step further by offering a way out. It formulated guidelines as to what would be a legally acceptable policy. The Court argued that the trend towards more restrictions to tobacco advertising in national regulations might lead to obstacles to the free movement of press products. 'In principle, therefore, a directive prohibiting the advertising of tobacco products in periodicals, magazines and newspapers could be adopted on the basis of article 100a' (para 98). A similar argument could be valid for certain forms of sponsorship. With these statements, the Court made an indirect contribution to policy-making [26] which strongly influenced the further course of the process. The problem-solving intervention of the Court opened the possibility for a new proposal by the Commission, introduced a year after the annulment [33].

The '2001' proposal Advertising for tobacco products in press and other printed publications are limited to publications intended exclusively for professionals in the tobacco trade and publications printed in third countries which are not principally for the Community market. Advertising not permitted in the press and other printed publications shall not be permitted on the internet. All forms of radio advertising and sponsorship shall be forbidden. Also other forms of sponsorship with cross-border effects shall be prohibited. Member states shall not prohibit or restrict free movement of products or services that comply with the directive.

The proposal had the same legal basis as the annulled Directive 98/43/EC (now, after amendment, article 95). However, the Commission made sure to emphasise the market-making element of the directive. Again the Commission reasoned that differences between national legislation result in barriers of free movement between member states for products and advertising related services. For sponsorship, distortion of competition was likely to increase. As member states were heading towards increasingly stringent advertising restrictions, harmonisation could only be logically based on a ban. However, this time the scope of the proposal was limited to advertising and sponsorship with a cross-border effect. Indirect advertising, as well as those elements of advertising with no cross-border effect, fell outside the scope of the proposal.

Whereas in the previous process the ENVI Committee was responsible for the report on the proposal, in this case the Committee on Legal Affairs and the Internal Market drafted the report for the EP [34]. It took 18 months to come up with 25 amendments, which according to members of the ENVI Committee, could 'lead to further annulment by the Court of Justice and which restrict the scope of the directive still further' [35].

The ENVI Committee, though not the prime responsible committee anymore, gave its opinion on the Commission proposal: 'the new proposal is much more modest in terms of protection of public health. (...) Studies have been carried out on the effects of partial or total advertising bans on the consumption of tobacco products. They have shown that partial advertising bans have only limited effects on smoking, since the industry can easily switch to advertising in other media. On the contrary, comprehensive bans on the advertising and promotion of tobacco products, covering all media and all uses of brand names and logos have proved to be significantly effective in reducing smoking' [34]. The ENVI Committee considered the Commission's proposal 'extremely disappointing from the point of view of health protection' [34], but the debate did not take place in a vacuum and the EU competence to adopt legislation should be taken into account. Therefore the proposal 'goes as far as possible. (...) Any amendment seeking to introduce new elements emphasizing the public health aspects would *de facto *undermine the appropriateness of the legal basis' [34].

During the debate in the EP [35], responsible Commissioner Byrne expressed his disappointment with the slow progress on the proposal: 'There have been delaying tactics, misinformation and, very often, misleading statements. It is a great pity that the Committee on Legal Affairs and the Internal Market was unable to work more quickly, in contrast to the much quicker Committee on the Environment, Public Health and Consumer Policy, whose members are the true experts in the EP. (...) I make no secret of the fact that I would have preferred to present a proposal for a complete ban (...). But, unfortunately, the legal framework does not allow us to go that far. (...) We have to recognise the legal constraints'.

In general, the discussion in the EP focused on the ECJ ruling and the question as to whether this new proposal indeed had a Treaty basis. For example, both German MEP Lechner (EPP) and Berger (PSE) argued that most publications simply had no cross-border effect. 'I believe this question of law to be eminently political in nature (...). We should be putting down a marker, and as legislators ourselves, respecting the powers of our counterparts in the national parliament, rather than again leaving this matter to the ECJ' (Lechner). However, in November 2002, the EP passed the watered-down proposal with 309 votes in favour, 203 votes against and 39 abstentions [36].

Once at the Council, agreement was reached on 2 December 2002 [33]. Again, two member states voted against the directive: the UK and Germany. Paradoxically, the UK had just passed a ban on tobacco advertising in November 2002. After a positive vote on the annulled Directive 98/43/EC, the UK voted against Directive 2003/33/EC, claiming that the text was too weak. It remains unclear whether this is the real argument, or whether the British government wanted to please some domestic constituents, with the knowledge that the directive would be adopted anyhow [1].

#### European Court of Justice ruling on Directive 2003/33/EC

After the adoption of Directive 2003/33/EC, the German government asked the ECJ to annul articles 3 and 4 [37], covering a ban on advertising in the press and printed publications, except those intended exclusively for professionals in the tobacco trade and printed in third countries not principally intended for the Community market; a ban on advertising on the internet for those advertisements that are not allowed in press and printed publications; and a ban on radio advertising and sponsorship.

The German government put forward five pleas in law in support of its action: article 95 (previously article 100a) was not the appropriate legal basis; the directive was adopted in breach of article 152(4) (previously article 129(4)). In addition, Germany also argued that there was a breach of duty to state reasons, of the rules governing the co-decision procedure, and infringement of the principle of proportionality. Whereas Directive 98/43/EC was only challenged on its legal basis, it seems that in this case, Germany searched for additional reasons to strengthen its position. However, in the light of settled case law, these grounds are very difficult to succeed.

Firstly, the German government held that none of the prohibitions laid down in article 3 and 4 of the directive contributed to eliminating obstacles to the free movement of goods, or to removing appreciable distortions of competitions. It claimed that there was hardly any cross-border trade of printed publications, consultation on the internet of printed publications from other member states was marginal and the limited range of transmitters implied that radio programmes addressed to the public in a locality or region could not be picked up elsewhere. 'The true purpose of those prohibitions is not to improve the conditions for the establishment and functioning of the internal market, but solely to protect public health' (para 24). The EP and the Council, supported by Spain, Finland, France and the Commission, disputed all these arguments.

The Court examined whether this new directive conformed to the criteria set in the previous case on Directive 98/43/EC. The three determining factors used to decide whether the new directive was legitimately based on article 95 included the need to harmonise, a favourable internal market purpose and a favourable internal market effect. The possibility that the directive also had a public health protection goal did not matter as long as there was also a market-making element. The Court used the same wording as in the previous case (see para 88): 'Provided that the conditions for recourse to article 95 EC as a legal basis are fulfilled, the Community legislature cannot be prevented from relying on that legal basis on the grounds that public health protection is a decisive factor in the choices to be made' (para 39).

The Court argued that disparities between national laws on tobacco advertising did indeed exist and resulted in legal obstacles to trade. The Court then examined whether article 3 and 4 were in reality designed to eliminate or prevent obstacles to the free movement of goods, the freedom to provide services or to remove distortions of competition. Given that article 8 of the directive prevents member states from prohibiting or restricting free movement of products and the freedom to provide services that comply with this directive, 'article 8 of the directive gives expression to the objective laid down in article 1(2) of improving the conditions for the functioning of the internal market' (para 74). Therefore article 95 was the correct legal basis of the directive and the Court dismissed this plea.

Germany also maintained that article 152(4) laying down the prohibition on any harmonisation of the laws and regulations of the member states in the field of public health was circumvented, because the true purpose of the directive was public health. The Parliament and the Council argued that that the conditions for recourse to article 95 were fulfilled and that this could not prevent them from taking measures that also had an impact on the protection public health. Since the Court had already ruled that the directive had the correct legal basis and explained in paragraph 39 that this directive could have both a market-making and market-correcting effect, the second plea was dismissed as well.

Thirdly, the German government asked for annulment, arguing that the requirement to show clearly and unequivocally the reasons for the measures adopted, as laid down in article 253 of the Treaty, were not fulfilled. However, the Court disagreed and stated that the 'recitals clearly disclose the essential objective pursued by the Community legislature' (para 114). The fourth argument concerned infringement of the co-decision procedure. Germany stated that amendments were made by the Council to article 10 and 11 of the directive without approval of the EP. However, the Court determined that 'by the present action, the applicant seeks to call into question the validity of articles 3 and 4 of the directive alone' (para 124), therefore, this plea necessarily needed to be dismissed. Finally, Germany tried to show that the prohibitions of article 3 and 4 'seriously compromise fundamental rights in the economic sectors concerned' (para 130), referring to the freedom of the press and of expression. The Council and the Parliament explained that these fundamental rights may 'be subject to certain restrictions or penalties, prescribed by law, which are necessary in a democratic society in the interests of the protection of health or morals' (para 141). The ECJ ruled that article 3 and 4 'may be regarded as measures appropriate for achieving the objective they pursue' (para 146) and therefore also dismissed this plea. Thus, Germany lost its appeal for annulment of articles 3 and 4 of Directive 2003/33/EC.

## Conclusion

The question why it took so many years of negotiation before the EU adopted a policy measure that could, in fact, improve the health situation in the Community, can only be answered by focusing upon the political sphere [5]. This article presented an analysis of the impact of the legal basis on the policy-making process. Following our framework of actor-centred institutionalism, actors' strategic behaviour is related to both their preferences and the institutional structure constraining or enabling them.

The policy-making process featured a high conflict level from its initial stage forward, not only because of differing views upon the impact of the ban upon smoking behaviour, but also, and more importantly, because of distributive conflicts. At a deeper level, the political difficulties reflected a conflict between public health and economic interests. As we showed in our analysis, in the case of tobacco advertising, the discussion on the legal basis was used to circumvent debate on how to balance public health and economic interests.

When the Commission came up with its proposal for a tobacco advertising ban in 1991, it had to play the treaty-base game in order to create a proper legal basis. The Treaty did not provide the Community with the legal competence to harmonise national regimes to attain a public health goal. One option was to use article 235, but this route had a serious drawback. Not only did it reduce the role of the EP to consultation, but it would also require unanimity in the Council, which would be politically unfeasible given the diverging preferences of the member states. The only option left was article 100a, which would require a qualified majority in the Council.

The assessment of the proposals' legal basis by the member states and the EP varied with their preferences. For a long time, achieving a qualified majority in the Council was impossible, because several member states formed a blocking minority. They mainly argued that the legal basis was incorrect. They tried to reduce the scope of the proposal and suggested an alternative legal basis that they knew in advance would never work, because of the required unanimity. Thus, opposing member states within the Council tried to change the problem-solving capacity of the EU from QMV to unanimity. However, after a new UK government took office, the Council adopted a common position.

In the EP, opposing MEPs tried to change the legal basis by asking the Council and the Commission questions. They also tabled amendments to change the legal basis. If the EP had indeed amended the Council's common position, the co-decision procedure would have resulted in a conciliation procedure between the EP and the Council. The actors involved were all aware that conciliation would most likely fail and result in a non-agreement. In the end, however, the EP adopted the common position without amendments.

Our case also illustrates the prominent role of the ECJ, not only in legal terms, but also in terms of policy-making. Asking the ECJ to annul Directive 98/43/EC because it had no correct legal basis was obviously a clear strategic choice by Germany and a few other dedicated opponents. This implied that the institutional setting changed from the mode of joint decisions to the mode of hierarchical direction. The ruling was remarkable, because the Court did not confine itself to the conclusion that the Treaty missed a proper legal basis for the ban. The ECJ went a significant step further by suggesting a way out. The Commission seized the opportunity offered by the ECJ and tabled a new proposal. In 2003, a new directive with a limited scope was adopted. This directive 'survived' after the ECJ dismissed the German challenge in December 2006.

The ECJ has been relatively generous in drawing the boundaries of the internal market treaty basis, including cases concerning the use of this basis for measures with perhaps more than an incidental health protection aim. Whereas the Court departed from this generous construction of article 100a as the legal basis in its ruling on Directive 98/43/EC [38], it ruled that the article was the valid legal basis for Directive 2003/33/EC.

Our case clearly demonstrates that the Commission strategically used its right of initiative. Despite the consistent ruling of the ECJ that the choice of a legal basis should always be a matter of legal principle [12], the strategy of the Commission reflected a high degree of political pragmatism. The choice of the legal basis evolved as a strategic game in itself: it became a permanent contentious issue throughout the policy-making process. The process was characterised by repetition of arguments that continued until the final stage. Beyond the question about the political will to adopt a ban on tobacco advertising, there was the juridical question as to whether the EU was in fact allowed to adopt such a measure.

The market-making versus market-correcting discussion is related to a broader question, namely the scope of EU health regulation in respect to the member states. In fact, the policy-making process on a tobacco advertising ban is related to the 'constitutional' foundations of health policy legislation in the Community.

EU health policies often have to be legitimised by Treaty articles that have no, or only an indirect link, to health and healthcare, such as the internal market treaty basis. However, the absence of a clear cut legal basis for health policies does not imply that the EU's impact on health is negligible. The single market exposes almost all areas of economic importance to competition, including goods and services related to health(care). In many cases, market-making interventions touching upon health – including other tobacco-control measures and regulations on the safety and quality of pharmaceuticals, food and medical devices – also protect consumers. These measures thus combine an internal market rationale with consumer protection and public health goals [39]. As long as the internal market, on its own, constitutes a sustainable legal basis, a health rationale can be central to internal market legislation. Thus, a market-correcting intervention is possible as long as it also has a market-making element. In the case of tobacco-control measures, this has resulted in significant action against smoking related morbidity and mortality [10]. So even though the EU has narrow direct competences in the field of health, European health policy is gradually and incrementally taking shape [40] based on other legal bases.

## Abbreviations

*CECCM *Confederation of European Community Cigarette Manufacturers; *EAC *Europe Against Cancer; *ECJ *European Court of Justice; *EEC *European Economic Community; *ENVI *Environmental, Public Health and Consumer Protection Committee; *EP *European Parliament; *EPP *European People's Party; *EU *European Union; *MEP *Member of the European Parliament; *QMV *Qualified Majority Voting; *SEA *Single European Act; *TEU *Treaty on European Union; *UK *United Kingdom

## Competing interests

The author(s) declare that they have no competing interests.

## Authors' contributions

SB collected, analysed and interpreted policy documents and conducted the interviews. HM revised the manuscript. All authors read and approved the final manuscript.

## Appendix: interviews April 2005 – March 2006

#1 Former civil servant German Ministry of health, 26 April 2005

#2 Former representative BASP, 18 May 2005

#3 Civil servant Dutch Ministry of Health, 1 June 2005

#4 Dutch civil servant health working group, 22 June 2005

#5 Former representative EPHA, 19 July 2005

#6 Dutch member of the European Parliament, 3 November 2005

#7 Former French civil servant health working group, 23 August 2005

#8 Former British employee of the ENVI Committee, 4 November 2005

#9 Civil servant Dutch Ministry of Health, 8 March 2006

## Pre-publication history

The pre-publication history for this paper can be accessed here:


